# Time course of the rubber hand illusion–induced analgesia

**DOI:** 10.1097/PR9.0000000000001252

**Published:** 2025-03-11

**Authors:** Benjamin Mosch, Xaver Fuchs, Theresia Tu, Martin Diers

**Affiliations:** a Clinical and Experimental Behavioral Medicine, Department of Psychosomatic Medicine and Psychotherapy, LWL University Hospital, Ruhr University Bochum, Bochum, Germany; b Department of Psychology, University of Salzburg, Salzburg, Austria

**Keywords:** Rubber hand illusion, Pain modulation, Embodiment, Visual-thermal integration

## Abstract

**Introduction:**

Previous investigations on pain modulatory effects of the rubber hand illusion (RHI) yielded mixed results. However, these studies used separate stimuli to induce pain and the RHI. Using a visual-thermal stimulation approach, the illusion-inducing stimulus was simultaneously the pain stimulus which ensured that participants focused entirely on the illusion-inducing stimulus.

**Objectives:**

In this study, we investigated the time course of pain modulation induced by illusionary body ownership over artificial hands using the *visual-thermal* RHI and the influence of the stimulation intensity.

**Methods:**

In a 2 × 4 within-subject design, participants received thermal stimulation on their hidden real left hand, while the rubber hand synchronously lit up red. Four stimulation intensities were used: moderate pain (+0°C), −0.75°C, +0.75°C, and +1.5°C. For control trials, the rubber hand was rotated by 180°. With the right hand, participants provided continuous pain ratings using a slide knob.

**Results:**

Embodiment ratings were higher in the RHI compared with the control condition. Continuous pain ratings were lower in the RHI condition for all temperature levels except for +0.75°C. Rubber hand illusion–induced pain reduction was observed throughout most of the stimulation interval, absent only at the very beginning and end.

**Conclusion:**

These findings suggest that visual-thermal induction of the RHI is consistently associated with increased embodiment ratings, regardless of the temperature level presented. The illusion is further accompanied by reduced pain ratings throughout major parts of the stimulation interval. On the whole, these findings speak for the robustness of the effect and the practicality of our visual-thermal stimulation approach.

## Abstract 2

Induction of the rubber hand illusion leads to higher embodiment ratings and pain reduction at almost all temperature levels, throughout most of the stimulation interval.

## 1. Introduction

The rubber hand illusion (RHI), first described by Botvinick and Cohen,^7^ is a common and robust method to induce transient changes in body representations: Watching a rubber hand being stroked with a brush while the own hidden hand is stroked synchronously can induce the feeling that the rubber hand belongs to one's own body (ownership of an artificial limb). It has been assumed that the mechanism behind this illusion involves multisensory integration of visual, tactile, and proprioceptive information associated with activity in multisensory areas, such as the premotor cortex.^13^

Previous research has investigated whether the RHI modulates the perception of pain but has yielded mixed results. Most studies reported decreased pain sensitivity when using “healthy” artificial hands ^8,14,16,18,29,31,41^; but increased pain sensitivity has been observed as well,^37,41^ whereas other studies found no effect on pain sensitivity whatsoever.^30^

These mixed results may occur due to a variety of mechanisms potentially underlying the RHI^21^ and the extent to which they are involved within a particular experimental RHI induction. Indeed, there is a large variability of experimental approaches used for the RHI.^36^ Regardless of the variability in experimental approaches, one aspect is consistent in almost all of the previously reviewed studies: the stimulation inducing the illusion and the pain stimulus were separated in terms of time and/or location. Consequently, participants had to attend 2 stimuli—the RHI-inducing stimulus and the pain stimulus—demanding them to distribute their attentional resources.

We recently introduced a variant of the RHI that was based on *visual-thermal* stimulation^40^ and adapted this method to induce the RHI with painful heat stimuli.^8^ Different from previous studies, the visual-thermal RHI provides the possibility to induce the RHI with the same painful stimulus that is rated by the participants. Accordingly, our new variant of the RHI ensures that full attention is focused on both illusion-inducing and pain-related aspects of the stimulus.

Using this paradigm, we found an analgesic effect of the heat-pain-induced RHI, presumably because this variant did not require participants to split their attention between the separate illusion-inducing and painful stimuli.^8^ Notably, previous studies on RHI-induced analgesia have mostly investigated pain intensity through retrospective assessments, whereas in this study we used continuous ratings that are recorded in real time during stimulation. These continuous ratings enable us to map the observed effects over time. In addition, possible distortions because of a delayed evaluation can thus be avoided.

So far, there have been no studies on the time course of pain perception during the RHI nor on the influence of the stimulus intensity. A better understanding of the RHI-induced pain modulation can be obtained by observing how the perceived pain intensity is modulated within a trial and over a series of trials within a condition. Such findings could provide information on underlying mechanisms like pain adaptation. For example, during tonic heat pain stimulation, the perceived pain level can increase because of temporal summation processes or decrease because of adaptation processes such as habituation. These processes are altered in chronic pain patients who sensitize more strongly when exposed to tonic heat pain.^22^ If the RHI-induced analgesia interacts with such effects, for example by counteracting sensitization, this would give valuable insights about the potential applicability in chronic pain treatments. In addition, better knowledge of the pain intensities that could be modulated with the RHI would provide valuable new insights into this kind of pain modulation. An analgesic effect of the RHI has so far been shown for moderate pain stimuli as well as for stimuli at the individual pain threshold. We aimed to extend these findings and anticipated analgesic effects across 4 different stimulus intensities below, at, and above moderate pain levels.

## 2. Materials and methods

### 2.1. Participants

Thirty-four healthy, right-handed participants (27 female and 7 male participants; mean = 24.03 years; SD = 7.34) took part in the experiment. There were no missing data for the pain intensity and unpleasantness ratings. In 5 participants, at least 1 question of the first 3 items of the embodiment questionnaire were missing in 1 block of 1 temperature condition; in 1 participant, 1 item was missing for both blocks of the +0° inverse control condition. Accordingly, 7 embodiment scores (∼1% of the data) were coded as missing and remained in the data set as the statistical model (see below) can handle missing data.

Simulation-based power analyses based on previous data acquired using a similar induction method^8^ revealed that a study with a sample size of 34 or more participants has a power of above 0.9 to detect an analgesic effect of the RHI (see https://osf.io/23pvm/). All participants were right-handed according to the Edinburgh Handedness Inventory,^32^ did not suffer from depression, as assessed by the German version of the Center for Epidemiological Studies Depression Scale,^17^ and have not been diagnosed with any chronic pain disorder.

Participants were recruited at the Ruhr University Bochum by advertisement on bulletin boards and social media. Inclusion criteria were right-handedness and an age above 18 years. Exclusion criteria were acute depression as well as chronic pain disorders. Participants were recruited, and data were collected between October 2019 and June 2021.

Participants received verbal instructions about the procedures and were informed they could stop the experiment at any time. Informed consent was obtained, and the study was approved by the local ethics committee of the Ruhr University Bochum (approval number: 17-6007) and adhered to the Declaration of Helsinki. The experiment took place in the Clinical and Experimental Behavioral Medicine laboratory.

### 2.2. Experimental design

During the experiment, we used warm or painfully hot temperatures on the participant's left hand (thenar) and red light on the rubber hand to evoke an RHI. The thermal and visual stimuli were presented synchronously. We used a 2 × 4 within-subject design with 2 different rubber hand orientations (factor RUBBERHAND: normal vs inverse) and 4 different temperatures (factor TEMPERATURE: +0°C, −0.75°C, +0.75°C, and +1.5°C, see “Determination of stimulus intensity”). In normal RHI trials, the rubber hand was presented in the same orientation as the real hand to invoke the RHI, whereas in inverse RHI trials, the rubber hand was presented in an anatomically impossible position, rotated 180°, to prevent the RHI.^13,20^ The experiment consisted of 2 blocks with every condition presented once in each block in randomized order.

### 2.3. Apparatus

**Figure 1.**
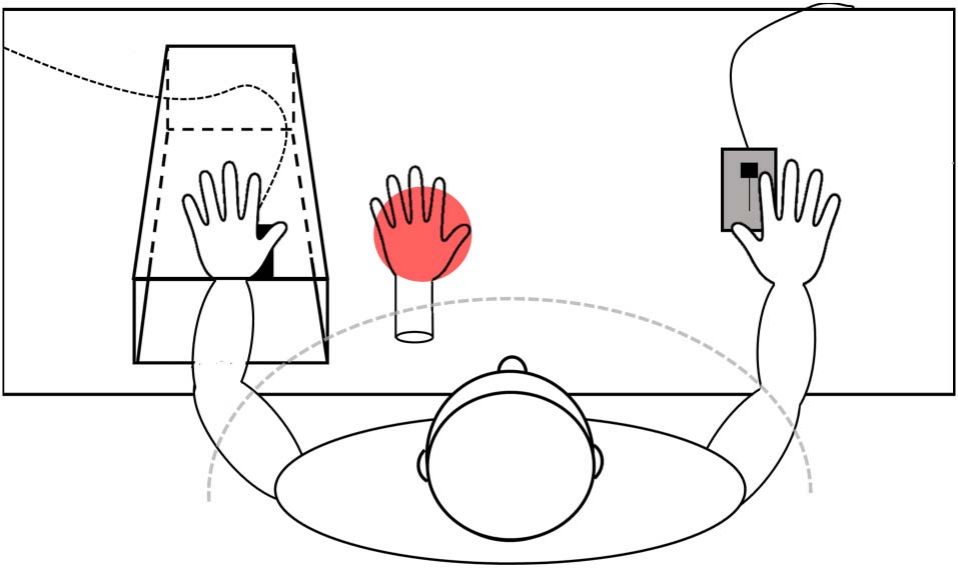
Experimental set-up. The left real hand's thenar was placed on the heat contact of the thermode and was covered with a wooden box. The right hand was placed approximately at the same height and distance as the left hand on a slide control knob, with approx. 30 cm from the body midline and 60 cm between the 2 hands. The rubber hand was located in front of the participant next to the wooden box, with its lower end covered by a cloak in normal RHI trials (grey dotted line). In inverse RHI trials, the rear end of the rubber hand was openly visible. The cloak on the front of the body was also used to cover the participants' arms. Red light was mirrored through a small hole in the table to illuminate the rubber hand from underneath, making the rubber hand light up in synchrony with the rising temperature of the thermode. RHI, rubber hand illusion.

The experimental setup is shown in Figure 1 and is similar to the one used in the study by Cordier et al.^8^

During the experiment, the participants sat on a chair at a table with their arms resting and palms facing downwards. They wore a black cloak to mask their arms and the proximal end of the rubber hand. The real left hand's thenar was placed on the heat contact (25 mm × 50 mm) of a thermode (Somedic SenseLab MSA, Sösdala, Sweden), which was inserted into the tabletop and covered with a box, so it could not be seen. The right hand was placed approximately at the same distance (approximately 30 cm from the body midline) as the real left hand and rested on a slide control knob that was used to rate the momentary pain level throughout the experiment (see ratings of pain and ownership). The rubber hand was a life-sized and sex-matched prosthetic limb with naturalistic shape, color, and texture (Otto Bock, Duderstadt, Germany) located in front of the participants, displaced 13 cm to the left of the body-midline. There was a distance of 15 cm between the index fingers of the real left hand and the rubber hand. Red light, emitted by a projector (InFocus IN26 Projector; InFocus International B.V., Lake Oswego, OR) under the tabletop, was mirrored through a hole in the tabletop and illuminated the rubber hand from underneath. The baseline color was black (RGB: 0; 0; 0) and the target color was red (RGB: 255; 0; 0). The red light emitted by the projector was presented at full intensity in synchrony with the thermode reaching the peak temperature. The experiment was controlled using PsychoPy v3.1.2.^33^

### 2.4. Determination of stimulus intensity

At the beginning of the experiment, the individual pain threshold was determined. First, the temperature was gradually increased (1°C/s) from a starting temperature of 30°C until the participants stopped the stimulation at an intensity perceived as just painful. The intensities of the last 3 of 5 ascending stimulations were averaged. In a second step, the calculated intensity was presented to the participants (analogously to the presentation in the following experiment) and adjusted in PsychoPy until verbally rated between 5 and 7 on a scale between 0 (no pain) and 10 (strongest pain). The determined temperature was set as the intensity eliciting moderate pain. For the sake of simplicity, we will hereafter refer to this temperature level as +0°C. In the experiment, 4 different target temperatures were used for RHI trials: individual nonpainful (−0.75°C) and different painful levels (+0°C, +0.75°C, and +1.5°C).

### 2.5. Rubber hand illusion stimulation procedures

In all conditions, the thermal stimuli (left hand) and the red light on the rubber hand were presented simultaneously with a synchronous waveform. The temperature started at a baseline temperature, set to a value 3°C below individual moderate pain (+0°C). Each trial started with the baseline temperature for 1 second. Then the temperature increased linearly to reach the target temperature within 2 seconds. The target temperature was held for 5 seconds, then the thermode returned to baseline within 2 seconds. One trial therefore lasted for 10 seconds. The first second of each trial was excluded for the analysis because some participants were still busy returning the slider to the desired position. After each trial, there was a variable intertrial interval lasting between 500 and 1000 milliseconds (uniform distribution). The same waveform was used for the red light presentation. During each condition, a continuous heat pain intensity rating was performed with the right hand. After each condition, the perceived heat pain intensity and ownership of the RHI were rated as well (see Table 1 and section “ratings of pain and ownership”). Each experimental condition was presented in each of the 2 blocks and consisted of 10 trials each (20 trials in total), yielding a total of approximately 30 minutes plus some additional time for completion of the questionnaires.

**Table 1:** Rubber hand illusion experience questionnaire.

During the experiment there were times when
(1) It seemed as if I was feeling the cooling/warming of the thermode where I saw the rubber hand lighten up
(2) It seemed as though the cooling/warming of the thermode was caused by the lightening of the rubber hand
(3) The rubber hand felt as if it was my own hand
(4) I felt as if my real hand was drifting to the right (towards the rubber hand)
(5) It seemed as if I might have more than 1 right hand or arm
(6) It seemed as if the cooling/warming of the thermode came from somewhere between my own hand and the rubber hand
(7) It felt as if my real hand was changing into rubber
(8) It appeared (visually) as if the rubber hand was drifting towards the left (towards my hand)
(9) The rubber hand began to look like my own (real) hand in *terms of shape, skin colour, freckles, or other features

Adapted from Botvinick and Cohen (1998) and previously used in Cordier et al. (2020).

### 2.6. Ratings of pain and ownership

During each condition, participants continuously rated the perceived stimulus intensity using a custom slide control knob with their right hand to follow the perceived pain intensity during stimulation.^43^ The slider consisted of a linear potentiometer whose resistance values were transformed to a range between 0 and 200 by a microcontroller (Arduino Nano; Arduino, Turin, Italy), sending these values to the computer using USB connection. The slider could be moved from the bottom (no sensation; value 0) to the top (strongest pain; value 200) crossing a haptically noticeable midpoint (just painful; value 100) that served as reference.

After each condition (ie, 10 trials), participants rated the painfulness and unpleasantness of the stimulus applied to the left hand using a visual analogue scale (VAS) in paper and pencil form. The VAS pain scale was presented as a 100-mm horizontal line anchored with “no sensation” (left) and “strongest pain” (right). The center of the line was labeled “just painful.” The VAS unpleasantness scale was also presented as a 100-mm horizontal line, anchored with “very unpleasant” (left) and “very pleasant” (right). The center was labeled “neutral.”

The ownership experience was assessed using a questionnaire adapted from Botvinick and Cohen^7^ and previously used by Cordier et al.^8^ (Table 1). The first 3 questions indicated the illusion intensity (target), and the mean was subsequently used as the embodiment score. The following 6 items examined the experienced sensation (distractor) during the illusion. The items were answered on a numerical rating scale from 0 (not at all) to 10 (most intense).

### 2.7. Statistical analyses and data processing

We used linear mixed models (LMMs) for statistical analyses. Visual inspection of residual plots did not reveal obvious deviations from homoscedasticity or normality showing that the data were suitable for analyses with LMM. The level of significance was set to *P* < 0.05.

Our primary aims were to assess the differential effects of RUBBERHAND and TEMPERATURE (experimental factors) on body ownership and pain experience (dependent measures). The ratings of body ownership, pain intensity and unpleasantness, as well as the continuous pain ratings were analyzed using 1 LMM for each measure. In each analysis, we used RUBBERHAND (normal or inverse) and TEMPERATURE (−0.75°C, +0°C, +0.75°C and +1.5°C) as fixed within-subject factors. We included participant as a random intercept factor, allowing for interindividual variability of intercepts. For the continuous pain rating, we included TIME as an additional factor with 9 levels, corresponding to 9 time bins of equal length distributed over 9 seconds (2-second ramp, 5-second plateau, 2-second ramp). We followed up significant effects in the LMMs using post hoc tests. *P* values were adjusted for multiple testing using the false discovery rate. For the comparison between normal and inverse RUBBERHAND conditions, we used 2-sided post hoc tests.

All statistical analyses were conducted using the R environment^35^ with the RStudio integrated development environment (RStudio Inc., Boston, MA). LMMs were performed with lme4 and lmerTest packages^3,23^; graphics were created using the ggplot2 package.^45^ Post hoc tests were carried out using the emmeans package for R that allows computing statistical comparisons between model's estimated marginal means.^24^

## 3. Results

The average stimulation intensity for the pain threshold was mean = 43.75°C (±3.71). For the adjusted stimulation values (+0°C), which correspond to a VAS rating between 5 and 7, the mean value was mean = 46.11°C (±2.17).

### 3.1. Continuous pain ratings

The LMM indicated significant main effects for the factors RUBBERHAND (*F*(1,112030.5) = 205.75, *P* < 0.001), TEMPERATURE (*F*(3,35.1) = 72.37, *P* < 0.001), TIME (*F*(19,111978.0) = 8530.16, *P* < 0.001), as well as the interactions RUBBERHAND × TEMPERATURE (*F*(3,111850.2) = 43.3, *P* < 0.001), RUBBERHAND × TIME (*F*(19,111978.0) = 4.07, *P* < 0.001), TEMPERATURE × TIME (*F*(57,111978.0) = 113.29, *P* < 0.001), and RUBBERHAND × TEMPERATURE × TIME (*F*(57,111978.0) = 2.12, *P* < 0.001).

**Figure 2.**
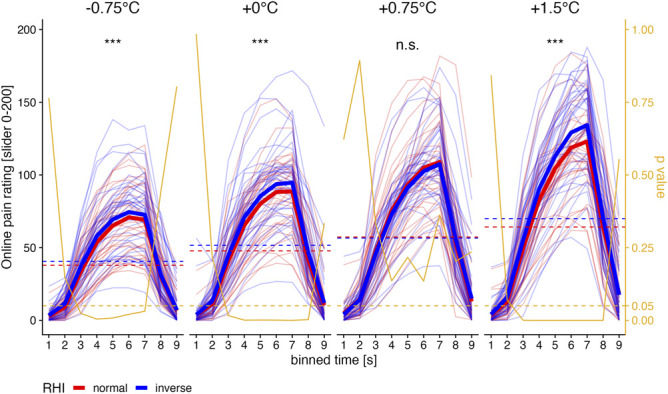
Time course of continuous pain ratings illustrated for all temperature levels and RHI orientations. The values on the *x*-axis represent a binned time variable. That means that corresponding *y* values are averages for the given time interval (ie, the time × value 2 means that values between second 1 and 2 were averaged). On the *y* axis, online pain ratings are illustrated from 0: no sensation to 200: strongest pain, crossing a midpoint at 100: just painful. Fine colored lines represent the mean ratings of each participant. Bold lines illustrate the estimated marginal means derived from the linear mixed models (see text for details). The dashed lines represent the mean ratings throughout the sum of all trials and time bins. The statistical annotations represent the post hoc tests comparing the conditions over all bins. Fine yellow lines represent the *P* value for the statistical post hoc test comparing the rubber hand conditions for the given time bin. The dashed horizontal yellow line represents the 0.05 significance level. ***P < 0.001; n.s., not significant; RHI, rubber hand illusion.

Post hoc tests for the interaction RUBBERHAND × TEMPERATURE revealed significant differences for all temperatures except for +0.75°C (−0.75°C: *P* < 0.001, +0°C: *P* < 0.001, +0.75°C: *P* = 0.102, +1.5°C: *P* < 0.001) with lower pain ratings for the normal rubber hand direction (Fig. 2). This interaction varied across time bins (expressed by the RUBBERHAND × TEMPERATURE × TIME interaction).

Post hoc tests for the interaction RUBBERHAND × TIME revealed significant differences for all time bins between 2 and 8 of the total 9 bins (*P* between <0.001 and 0.029, see https://osf.io/23pvm/).

### 3.2. Body ownership

**Figure 3.**
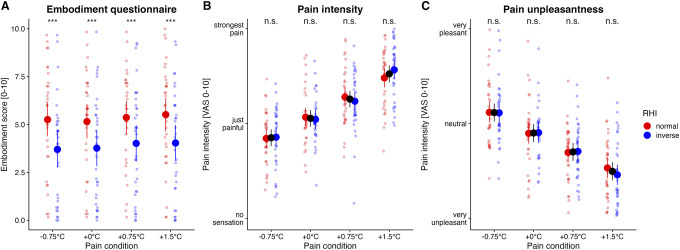
Embodiment (A), pain intensity (B), and unpleasantness ratings (C) shown according to the respective temperature level and RHI orientation. The individual ratings are shown as small dots. Vertical lines indicate 95% confidence intervals. ***P < 0.001; n.s., not significant. Note that there were no significant effects of RUBBERHAND for the pain intensity and unpleasantness ratings. The estimated marginal mean values for the factor RUBBERHAND derived from the linear mixed models are shown for descriptive reasons in panels (B) and (C). RHI, rubber hand illusion.

The linear mixed model showed a significant main effect of RUBBERHAND on embodiment (*F*(1,33.1) = 22.77, *P* < 0.001). We found no effect of TEMPERATURE (*F*(3,463.3) = 2.01, *P* = 0.112), and there was no significant interaction between the factors (*F*(3,463.3) = 0.18, *P* = 0.911) (Fig. 3).

### 3.3. Pain intensity

The LMM indicated a significant main effect for the factor TEMPERATURE (*F*(3,503) = 131.520, *P* < 0.001) and no effect for RUBBERHAND (*F*(1,503) = 0.0937, *P* = 0.76) or the interaction between the factors (*F*(3,503) = 1.3, *P* = 0.274) (Fig. 3).

### 3.4. Pain unpleasantness

The LMM indicated a significant main effect for the factor TEMPERATURE (*F*(3,503) = 104.245, *P* < 0.001) and no effect for RUBBERHAND (*F*(1,503) = 0.329, *P* = 0.566) or the interaction between the factors (*F*(3,503) = 0.555, *P* = 0.645) (Fig. 3).

## 4. Discussion

This study shows, for the first time, the detailed time course of RHI-induced analgesia for 4 temperature levels. We found that pain reduction was perceived at an early stage of the temperature increase and throughout most of the recorded time bins. Beyond that, we replicated the analgesic effect of the RHI using continuous pain ratings collected in parallel with the stimulation. Our findings represent an extension of previous results on the effects of visual and body-related stimulation and body ownership on pain perception.

Watching the own body can reduce the perceived intensity of experimental pain,^12,26,27^ ameliorate chronic pain,^11,30,44^ and improve pain treatments.^5,25^ However, watching body parts does not always result in analgesia,^38^ and under certain conditions, it may even have detrimental effects.^19^ Similarly, the modulation of body ownership yielded mixed results.^14,16,18,29,31,37,41^

As in a previous study by Cordier et al.,^8^ we used the same stimulus to induce pain and the RHI. Different from that study, we recorded the pain rating simultaneously to the thermal stimulation using continuous ratings. The time bins 2 to 8 (which were within the ramp to and from the plateau) showed significantly higher pain ratings for the inverse compared with the normal rubber hand, indicating an analgesic effect of the RHI. In addition, mean ratings were significantly different with higher pain ratings for inverse compared with normal rubber hand orientation for −0.75°C, +0°C, and +1.5°C, supporting our previous finding of an analgesic effect because of the RHI.

Compared with most aforementioned studies that have explored the relationship between pain perception and the RHI, our experimental design allowed to use the same stimulus for thermal stimulation and induction of the RHI.^8,40^ In this way we could ensure that participants focused on aspects of the RHI and were not distracted by a separate heat stimulus and vice versa. In other words, this procedure allows participants to fully focus on the illusion-inducing stimulus and prevents splitting attention. Previous studies have emphasized the importance of attention processes for (neuronal) pain modulation. In this respect, it has been shown that corticofugal projections can modulate the responses of dorsal column nuclei to tactile stimuli.^28^ This could in turn contribute to the mechanisms of selective attention, according to Torta et al.^39^ A particularly important factor for modulation would therefore be the degree of attention devoted to relevant body parts.

### 4.1. RHI induction leads to decreased continuous pain ratings

In our previous investigation,^8^ we demonstrated that a start stimulus of 47°C, adjusted to be between 5 and 7 on a scale between 0 (no pain) and 10 (worst imaginable pain), was perceived less intense during synchronous compared with asynchronous stimulation for ratings assessed after the stimulation. Using continuous pain ratings in our present study, we can illustrate the time course of this analgesic process. In line with the results by Cordier et al.^8^ and other investigations that used post hoc ratings,^14,16,18,29,31^ experiencing the RHI led to decreased continuous pain ratings in our setting. Accordingly, we replicated the analgesic effect using a different control condition to the asynchronous simulation frequently used (but see also “Pain ratings after the stimulation are not positively affected through the RHI” for a discussion of the post hoc ratings). Interestingly, pain reduction was perceived almost during the entire stimulation (Fig. 2), except for the very start and end. Pain reduction begins as early as time bin 2 of each stimulation cycle, corresponding to only around 1.5 seconds after the start of the temperature increase. In addition, there was a fairly immediate decrease in continuous pain ratings with a decrease in stimulation intensity.

Pain reduction is perceived at almost all the chosen stimulation intensities (conditions −0.75°C, +0°C, and +1.5°C). However, it is not clear why there was no measurable effect at +0.75°C.

### 4.2. Rubber hand illusion–induced increase of body ownership at all temperature levels

On another note, we were able to induce a feeling of body ownership for a rubber hand using a combination of light and (non-)painful heat stimuli.^8,40^ In that respect, our analyses revealed a significant main effect of RUBBERHAND, but no effects for TEMPERATURE or the interaction between the factors. This illustrates that the RHI could be induced consistently and did not depend on the respective stimulus intensity. The observed mean ratings on the ownership questionnaire^7^ lie in a range between 5 and 6, which roughly corresponds to previous studies on the RHI that have used the same questionnaire.^4,8,15^

Several studies have suggested that the RHI can be induced particularly well through C-fiber affective touch.^9,10,42^ Beyond that, these studies also show that light touch seems to be especially suitable. In our experimental setup, the painful stimulation was also based on C-fiber affective input. However, in this case, stimuli were in the realm of painful thermal stimulation. Notably, induction of the RHI using painful stimulation differs from classical brush-stroking experiments regarding the (neuro-)psychological processing mechanisms involved because pain is mediated by the affective system.^1,6,34^ Our findings suggest that presenting more intense/painful stimuli does not have a significant impact on illusion strength. Thus, stronger affect in the sense of more painful stimulation does not necessarily lead to a stronger illusion. Arguably, the relationship between stimulation intensity and illusion strength is different for pain stimuli than for touch. As for touch, going from light to strong touch has been shown to diminish the RHI.^42^

For future studies, our findings indicate that different temperatures below, at, and above moderate pain levels could prospectively be used without having to expect that this could potentially diminish the RHI.

### 4.3. Pain ratings after the stimulation are not positively affected through the rubber hand illusion

In contrast to the results derived from the continuous pain ratings described above, LMM for pain intensity and unpleasantness ratings recorded after the stimulation showed no significant effects for RUBBERHAND. Accordingly, we could not demonstrate the previously reported analgesic effect in this part of our data.^8^ To better understand this null result, we scrutinized the main differences to our previous study. Apart from the continuous pain rating, the main difference was that the previous study used an asynchronous RHI control condition, whereas the current study used an inverted hand with synchronous stimulation. Although an inverted-hand control condition has some advantages, it is possible that effects are somewhat smaller using this procedure and not detectable in post hoc ratings. An advantage of the inverted control condition is that it avoids potential confounding effects of ownership and of the stimulation as such. In the present study, the stimulation is identical between the RHI and the inverted condition, and all differences in response to the stimulation must be attributed to the hand stimulus. When using an asynchronous control condition, some parts of the effects can be related to synchronous vs asynchronous stimulation, but independent of body ownership processing. It is possible that some proportion of the effect in previous studies is indeed attributable to stimulation types rather than body ownership. For example, Cordier et al.^8^ observed that synchronous stimulation had an analgesic effect when using a glass ball instead of a hand for which body-ownership processing is less relevant. One possibility is therefore that because the present study eliminates effects of stimulation type, the total effects are smaller. The online ratings might be more sensitive because they are free of memory biases and show the analgesic effects.

Finally, it should be mentioned that studies have shown an influence of continuous ratings on response behavior in retrospective ratings.^2^ Accordingly, in some cases, an online rating procedure might lead to the retrospective rating approaching the mean value of momentary pain. Whether such an effect exists in our ratings cannot be ruled out on the basis of the collected data. However, the influence should be of negligible importance for the statements we have made, especially because we are mainly referring to the online ratings.

## 5. Conclusions

The results described above expand our understanding of the versatile effects that visual and body-related stimulation as well as body ownership can have on the experience of pain. In this context, particular interest was given to the temporal course of pain perception. Continuous pain ratings demonstrated an analgesic effect through RHI induction. Interestingly, pain reduction was perceived almost during the entire stimulation phase, except for short intervals at the start and end of the stimulation. On another note, we were able to demonstrate that the RHI was consistently reflected in body ownership ratings, regardless of the respective temperature level in relation to the individual pain threshold.

## Disclosures

The authors have no conflicts of interest to declare.
